# Supplementary material for: Role of schools in community mobilisation to improve IYCF practices in 6–24-month-old tribal children in the Banswara district, India: findings from the qualitative PANChSHEEEL study
Source: BMJ Open. 2022 Apr 11;12(4):e047741. doi: 10.1136/bmjopen-2020-047741 (PMC9006840; doi:10.1136/bmjopen-2020-047741)
Supplement: Author's manuscript [file bmjopen-2020-047741.draft_revisions.pdf]

BMJ Open is committed to open peer review. As part of this commitment we make the peer review history of every article we publish publicly available.

When an article is published we post the peer reviewers' comments and the authors' responses online. We also post the versions of the paper that were used during peer review. These are the versions that the peer review comments apply to.

The versions of the paper that follow are the versions that were submitted during the peer review process. They are not the versions of record or the final published versions. They should not be cited or distributed as the published version of this manuscript.

BMJ Open is an open access journal and the full, final, typeset and author-corrected version of record of the manuscript is available on our site with no access controls, subscription charges or pay-per-view fees (http://bmjopen.bmj.com).

If you have any questions on BMJ Open's open peer review process please email info.bmjopen@bmj.com

BMJ Open

The Role of Schools in the PANChSHEEEL Project – an Approach to Identify and Address Factors Influencing Infant and Young Child Feeding Practices and Nutrition in India.

Journal:	*BMJ Open*
Manuscript ID	bmjopen-2020-047741
Article Type:	Original research
Date Submitted by the Author:	07-Dec-2020

Complete List of Authors:	Lakhanpaul, Monica; University College London, Institute of Child Health<br>Roy, Susrita; Save The Children, National Support Office<br>Lall, Marie; University College London, Institute of Education<br>Chaturvedi, Hemant; Save the Children<br>Khanna, Rajesh; Save the Children, National Support Office<br>Allaham, Shereen ; University College London Institute of Epidemiology and Health Care, Department of Epidemiology and Public Health; Aceso Global Health Consultants Ltd<br>Demel, Isabel-Cathérine; King's College London Faculty of Life Sciences and Medicine, Guy's, King's and St.Thomas' School of Medicine<br>Benton, Lorna; University College London Institute of Child Health, Population, Policy and Practice<br>Vijay, Virendra ; Indian Institute of Technology Delhi<br>Sharma, Sanjay ; University College London, Engineering for International Development Centre<br>Manikam, Logan; University College London Institute of Epidemiology and Health Care, Department of Epidemiology and Public Health<br>Santwani, Neha; Save The Children, National Support Office<br>Pattanaik, Satya Prakash ; Save the Children<br>Singh, Tol; Save the Children<br>Pandya, Pramod; Save the Children<br>Dang, Priyanka; Save the Children, National Support Office<br>Parikh, Priti; University College London, CEGE

Keywords:	PUBLIC HEALTH, Community child health < PAEDIATRICS, NUTRITION & DIETETICS, International health services < HEALTH SERVICES ADMINISTRATION & MANAGEMENT

SCHOLARONE™
Manuscripts

I, the Submitting Author has the right to grant and does grant on behalf of all authors of the Work (as defined in the below author licence), an exclusive licence and/or a non-exclusive licence for contributions from authors who are: i) UK Crown employees; ii) where BMJ has agreed a CC-BY licence shall apply, and/or iii) in accordance with the terms applicable for US Federal Government officers or employees acting as part of their official duties; on a worldwide, perpetual, irrevocable, royalty-free basis to BMJ Publishing Group Ltd ("BMJ") its licensees and where the relevant Journal is co-owned by BMJ to the co-owners of the Journal, to publish the Work in this journal and any other BMJ products and to exploit all rights, as set out in our licence.

The Submitting Author accepts and understands that any supply made under these terms is made by BMJ to the Submitting Author unless you are acting as an employee on behalf of your employer or a postgraduate student of an affiliated institution which is paying any applicable article publishing charge ("APC") for Open Access articles. Where the Submitting Author wishes to make the Work available on an Open Access basis (and intends to pay the relevant APC), the terms of reuse of such Open Access shall be governed by a Creative Commons licence – details of these licences and which Creative Commons licence will apply to this Work are set out in our licence referred to above.

Other than as permitted in any relevant BMJ Author's Self Archiving Policies, I confirm this Work has not been accepted for publication elsewhere, is not being considered for publication elsewhere and does not duplicate material already published. I confirm all authors consent to publication of this Work and authorise the granting of this licence.

For peer review only

The Role of Schools in the PANChSHEEEL Project – an Approach to Identify and Address Factors Influencing Infant and Young Child Feeding Practices and Nutrition in India.

Monica Lakhanpaul[1,2]
Susrita Roy[3]
Marie-Carine Lall[4]
Hemant Chaturvedi[5]
Rajesh Khanna[3]
Shereen Allaham[6,7]
Isabel-Catherine Demel[7,8]
Lorna Benton[9]
Virendra Kumar Vijay[10]
Sanjay Sharma[11]
Logan Manikam[6,7]
Neha Santwani[3]
Satya Prakash Pattanaik[5]
Tol Singh[5]
Pramod Pandya [5]
Priyanka Dang[3]
Priti Parikh[11]

Institutions

1. Population, Policy and Practice, UCL Great Ormond Street Institute of Child Health, 30 Guildford Street, London WC1N 1EH, UK
2. Whittington Health NHS Trust, London, UK, N19 5NF
3. Save the Children, National Support Office, Gurugram, India
4. Institute of Education, University College London, UK
5. Save the Children, Rajasthan, India
6. Department of Epidemiology and Public Health, University College London Institute of Epidemiology and Health Care, London, UK.
7. Aceso Global Health Consultants Limited, London, UK
8. Department of Life Sciences & Medicine, King's College London GKT School of Medical Education, London, UK
9. Great Ormond Street Institute of Child Health, University College of London, UK.
10. Indian Institute of Technology (IIT) Delhi, India
11. Engineering for International Development Centre, University College London, London WC1E 7HB

Corresponding Author:
Monica Lakhanpaul
Population, Policy and Practice, UCL Great Ormond Street Institute of Child Health, 30 Guildford Street, London WC1N 1EH, UK
Email: m.lakhanpaul@ucl.ac.uk
Telephone: 020 7905 2259

Word count: 4636

For peer review only - http://bmjopen.bmj.com/site/about/guidelines.xhtml

ABSTRACT

Objective: The PANChSHEEL project is targeted at developing locally feasible solutions for successful implementation of prescribed IYCF (Infant and Young Child Feeding) practices at individual, household, community and environmental level through a participatory approach across four themes: Health, Education, Engineering and Environment (HEEE). This study aims to understand both barriers and facilitators of practicing IYCF in rural India, to explore interventions for each variable and to identify potential stakeholders from the community to engage in participatory approaches. In this context, one intention is to explore the feasibility of schools in partnership with the Angawadi centres as innovation hubs to foster positive change within communities.

Design: 5-phase formative study; Phase 1 focused on identifying local feeding practices. Phase 2 involved identifying the local challenges, opportunities and needs for children in the first 6-24 months. Phases 3-5 focused on the analysis and intervention design, its review and dissemination.

Setting: This study was undertaken in nine villages within two blocks 'Ghatol and Kushalgarh', in the Banswara district of Rajasthan, India.

Participants: 17 schools were identified in the study area. Interviews were conducted with local opinion leaders and representatives in the education sector, including principals, school teachers, and Block and District Education officers.

Results: Schools were found to be crucial in capturing IYCF diversity and dynamics in communities studied. Teachers were well-respected locally for engaging with IYCF practices. During the feasibility testing, the information imparted to children via teachers was observed to have the potential to change IYCF practices of targeted parents and communities.

Conclusions: Schools have been shown to be an important setting for community mobilisation. Developing a supportive environment around schools, geared towards reinforcing healthy IYCF practices and utilising children as change agents hold potential for testing in future wider scale intervention studies.

Abstract word count: 291 words

KEYWORDS: Global Health, Nutrition&Dietetics, Community Child Health, International Health Services, Complementary feeding, IYCF practices, school-based interventions

For peer review only

Article Summary

Strengths and Limitations of this study

Strengths

- The PANChSHEEEL (Participatory Approach for Nutrition in Children: Strengthening Health Education Engineering and Environment Linkages - HEEE) project was an interdisciplinary study, designed to explore an array of important themes such as health, education, engineering and environment that influence Infant and Young Child Feeding (IYCF) practices and nutrition in India with the aim of developing a socio-culturally appropriate, tailored, innovative and integrated multisectoral HEEE Nutrition package to optimise IYCF practices for children aged 6-24 months.
- This study established the advantages of using schools to bring communities together and disseminate information regarding ideal IYCF practices whilst exploring the potential of working with children as community change agents.

Limitations

- Due to time and resource constraints, the study relied on the coverage and completeness of external data, e.g. a list of the children under the age of 2 years resident in the study area (prepared by AWW and/or ASHA), as a starting point for the data collection.
- The study covered only 9 villages in total across two blocks in the state of Rajasthan. Despite the main formative study being led across 18 villages, the phase of study directly related to testing the feasibility and acceptability of selected pilot school-based interventions were only conduced in 9 villages.

For peer review only

Introduction

Many low-and-middle-income countries (LMIC) have been grappling with the problem of malnutrition for decades. Due to its persistent and chronic nature, malnutrition affects a large proportion of the population in these countries. The triple burden of malnutrition presents itself in the form of stunting, wasting and anaemia - all of which are especially common in women and children.

It is well known that the period between 6 and 24 months of age represents a critical time for infant growth. In LMIC, stunting is generally the consequence of infectious disease and low nutrient intake, particularly inadequate energy and protein intake, relative to nutritional requirements [1]. In addition to infectious disease prevention strategies, supplementary Infant and Young Child Feeding (IYCF) interventions targeting this critical period are most effective in reducing malnutrition and promoting childhood growth and development [2]. Based on the evidence globally, achieving universal coverage of optimal breastfeeding could prevent 13% of global deaths occurring in children less than five years of age, while appropriate IYCF practices could result in an additional 6% reduction in under-five mortality [3].

Conscious of this fact, India introduced an 'Integrated Child Development Service' (ICDS) in 1975 to enhance nutrititional and health outcomes for pregnant women, breastfeeding mothers and children up to age 6. The programme has since been expanded to providing supplementary nutrition, immunisation and pre-school education across all Indian states. One of ICDS's flagship interventions is a centrally sponsored National Programme of Nutritional Support linked to Primary Education (Midday Meal Initiative), delivering hot cooked lunches to all the nation's school children to address the widespread problem of child under- and malnutrition. Another has been the introduction of rural child care centres, so called Anganwadi Centres (AWC): introduced by the Government of India (GoI), they are linked with schools and aimed at providing services and skilled staff to enhance child nutritional outcomes, basic medical aid and preschool education for 2-5year olds and their mothers in the community setting. Whilst there has since been a significant improvement in the care continuum from pregnancy to early childhood, households from the lowest quintiles of the economic strata and women with poor scooling levels, especially in the rural states with the highest burden of undernutrition, remain difficult to reach. [4]

The PANChSHEEEL (Participatory Approach for Nutrition in Children: Strengthening Health Education Engineering and Environment Linkages - HEEE) project is an interdisciplinary study, designed to explore HEEE factors that influence IYCF practices and nutrition in India. The study aimed to develop a socio-culturally appropriate, tailored, innovative and integrated cross sector HEEE Nutrition package to support optimal IYCF practices for children in rural India aged 6-24 months.

For peer review only - http://bmjopen.bmj.com/site/about/guidelines.xhtml

To implement the above approach in a cost-effective manner it is crucial to identify occasions and settings where community members gather at regular intervals with children. Since 1986, the World Health Organisation (WHO) has placed particular emphasis on the potential of health promotion strategies in a school setting [5], but the sustainability of school-based interventions remains relatively unexplored, especially in low- and middle-income countries [6].

Schools appear to be an important setting for implementing interventions, as they offer continuous, intensive contact with children. By contributing to students' health literacy and behaviours, school-based health promotion can be particularly valuable in the context of developing countries where low public health literacy and high burden of disease are faced. [7] Furthermore, children have been demonstrated to be change agents who are able to transfer information and play as an active-partner in the school-home relation [8]. During the era of Millenium Development Goals (MDGs) (nowadays known as 'Sustainable Development Goals' (SDGs)), school-enrolment and universal primary education have increased in developing countries, giving school-based interventions the potential to reach significant numbers of children [7]. School infrastructure and physical environment, hygiene campaigns, policies, curricula and teachers have the potential to positively influence physical, socio-cultural, and policy characteristics of the micro-environment [9]. Moreover, schools are well known to be used as a centre of change especially during crises in high-income countries [9] [10] as long as low- and middle-income countries [11] [12].

For this reason, schools were considered as a potential medium for information collection and dissemination for our intervention package (IP) especially due to the success of other school-based interventions using children as change agents [8]. In a literature review on school-based health promotion and their impact in developing countries, it was found that a majority of school-based interventions addressed nutrition, hygiene, oral health and communicable diseases [7], including nutrition education and hygiene awareness campaigns to reduce the incidence of worm infections. Implementation was carried out by schoolteachers, nurses and other community members, with study results suggesting an improvement in the knowledge and attitudes of their target population [7]. Furthermore, many school-based interventions have the advantage of being a cost-effective method to deliver interventions with a broad reach, enrolling participants from different socio-economic backgrounds, ages and at-risk groups [13].

In this paper, we aim to discuss the feasibility and acceptability of the IP which was disseminated, using the network of existing Anganwadi centres (AWC) and schools. We also explored the possibility and benefits of using schools as the change centres and the role of children as community change agents for our IP.

For peer review only - http://bmjopen.bmj.com/site/about/guidelines.xhtml

Methodology

Study design

The PANChSHEEEL project was a 5-phase formative study; Phase 1 focused on identifying and documenting local practices with regard to IYCF practices, sanitation, education & access to local resources (such as energy and water). The existing Government of India (GOI) initiatives in the study sites were also mapped during Phase 1. Phase 2 involved extensive fieldwork to identify local challenges, drivers, resources, opportunities and needs for children in the first 6-24 months of life at individual, household, community and environmental level. Phases 3, 4 and 5 focused on the analysis and mapping of HEEE linkages, intervention design and piloting, as well as review and dissemination phases of this project.

In this project, members from University College London (UCL), Institute of Child Health, Institute of Education and Faculty of Engineering Sciences, and expertise from; Save the Children- India, Indian Institute of Technology, New Delhi (IIT-Delhi) and Jawaharlal Nehru University, New Delhi (JNU) formed the Core Research Team (CT). The participatory approach actively involved local Community Researchers (CR), Community Champions (CC), and Community Members (CM) selected from the nine villages within Ghatol and Kushalgarh Block of Banswara district of Rajasthan (India). A mixed-methods approach structured by a socio-ecological framework was used for data collection at individual, household and community levels. Co-design of the IP was done through an iterative process.

In order to design the intervention, inputs were sought from stakeholders at various levels with four major information sources used in the process; (1) Community perspectives, (2) Review of policy and program environment, (3) Expert advice & (4) Good practice models of Non-Governmental Organizations. The purpose was to enhance the research teams' understanding of the nature of services and environmental conditions in each study villages, allowing them to tailor potential future interventions. Village profiles were created by the means of social mapping and community profiling as well as Transect walks, broad narrative discussions and seasonal income calendar. Interactions with identified CC and lay CM (e.g. mothers, fathers, and grandmothers), as well as other key male members of the household were also undertaken. Interactions at the village level were conducted with various levels of public health service providers, from Community Health Workers (Anganwadi Workers AWWs, Auxiliary Nurse Midwives ANMs) and local opinion leaders (e.g. school teachers), to policymakers at various levels (block, district, state, nation). To understand the multitude of factors that influence IYCF practices, a parallel mixed methods approach with both quantitative and qualitative elements was adopted. This paper focuses on the qualitative findings of the formative study using schools as a potential site for community engagement and intervention.

For peer review only

Schools selection

The formative research phase commenced after a systematic selection of nine study villages from the two blocks of Banswara, namely Ghatol and Kushalgarh. Out of the 18 villages proposed initially by 'Save the Children India', 9 were selected purposively based on an interaction of the Core Research Team with 1) the local community, 2) the teachers at the local elementary schools and 3) the Anganwadi workers and ASHA in each village. Schools where the relationship with the local village and functionality of the school itself were poor, e.g. low number of teachers and students, insufficient engagement with the study, were excluded. Secondly, villages where a lack of interaction between different social groups or lack of interest in the project was noted, were subject to exclusion. Finally, the linkage of the schools with the AWCs, e.g. the government-funded centres that provide facilities such as food, vaccines, medical treatments to 2-5-year olds alongside assisting mothers in accessing other government schemes/services, was a key determining factor. Schools, where the AWC was not directly associated with the school or where the AWW displayed insufficient interaction, were excluded. A summary of the inclusion and exclusion criteria for the study villages and schools is outlined in Figure 1. Overall, 17 schools were identified in the nine selected villages.

Figure 1. Inclusion and exclusion criteria for the study villages and schools

Patients and Public involvement and Engagement

Patient/Participant and Public Involvement and Engagement (PPIE) representatives were identified, selected and engaged in every step of the study from protocol development over study design to the dissemination of the results.

Community members from the nine villages within Ghatol and Kushalgarh Blocks of the Banswara district of Rajasthan were actively supporting the Core Research Team in the context of PANChSHEEEL's participatory approach. To help steer the project across its phases locally, Community Researchers (CR) from the study blocks Ghatol and Kushalgarh were recruited. The inclusion criteria encompassed (1) being educated, (2) having previous field experience in Banswara district, e.g. understanding the village context and social networks locally, and (3) being conversant in both 'Hindi' and 'Wagdi' languages (given that the most prevalent language dialect in the nine study villages is 'Wagdi'). With the help from the Save the Children' Team from the Rajasthan State Office, two suited individuals were identified. The Community Researchers were male adult members of the study population who understood the local village and social context, providing special insight about their communities and environment to the study. Both had shown satisfactory engagement in a previous project by 'Save the Children' and were local to the study blocks of Ghatol and Kushalgarh, and hence recruited on contractual basis during Phase 1 of the study. Their main role was to identify potential

For peer review only

candidates who could engage actively in project activities, as well as engaging with the Community Champions (CCs), who in turn helped establish linkages with the Community Members (CMs) and improve networks between schools, FLWs and villages. Additionally, two female Community Researchers were recruited for the data collection phase, since it was felt that mothers may be more comfortable responding to the questionnaire and reporting about breastfeeding and complementary feeding to female Community Researchers.

During the formative phase and stakeholder mapping workshop, relevant stakeholders were identified and recruited. Considering the possibility of snowballing, e.g. a sampling method where existing participants inform the recruitment of future participants from among their acquaintances and relevant to the study, the research team was open to any new stakeholder(s) identified during the data collection. Key participants were community members including mothers, paternal grandmothers, fathers/male members of the household, elected representatives from the village (e.g. FHWs viz. ANM, AWW and ASHA), Block Education officers, Ward Panches and the village school teachers and principals. All participants in the mothers' group had children below the age of two years; most of them also had an older school-going child. Similarly, all grandmothers had a grandchild under two years of age. The recruitment of participants was conducted based on availability and willingness to participate.

Data collection

Data collection at individual, household and community levels was carried out by the means of a mixed-methods approach structured by a socio-ecological framework. The qualitative data were collected using two methods: 1) Key Informant Interviews (KII) with AWWs, Accredited Social Health Activists (ASHAs), ANMs, Elected Panchayat Representatives (PRI members), school teachers and block education officers and 2) Focus Group Discussions (FGD) with mothers and grandmothers of children 0-2 years of age. The members of the research team conducting the qualitative data collection received a two-day training by a co-Investigator in Wagdhara, a non-profit organization based at Banswara.

Data was collected by the CRs with assistance from CCs for scheduling meetings with stakeholders. The CR were also trained intensively on techniques and tools to FGDs, KII and Broad Narrative Group Discussions. PowerPoint Presentations and handouts were used for training.

For peer review only - http://bmjopen.bmj.com/site/about/guidelines.xhtml

Topic Guide

Thematic guides were designed through literature review, expert consultation and evolved through pilot testing with different population sub-sets. Broadly speaking, the interviews covered four dimensions – 1) Health and Nutrition, 2) Education, 3) Water, Sanitation and Hygiene (WASH) and 4) Energy. This allowed the team to capture current practices across all four areas, as well as barriers and opportunities towards fostering a positive change within a school setting. The focus was on collecting data on the infant and young child feeding practices (IYCF) in the villages.

Data management and analyses

The qualitative data collected was transferred from the recording device to the encrypted laptop of the SCI Nodal Person. The research team also met at the end of each day to share the key observations and reflections about the data based on the notes taken during the KII and FGD.

Audio-recorded data was translated and transcribed alongside detailed summary of field notes prepared. Data from open-ended questionnaire questions were evaluated, discussed and summarised across the various IYCF themes for each intervention point. The initial data collected by Community Researchers (CR) further underwent respondent validation. Respondent validation was conducted in a workshop involving Community Champions (CC) facilitated by Community Researchers under the guidance of Save the Children staff in the month of September 2018. The steps of a thematic analysis were employed, e.g. identifying, analysing and interpreting patterns of meaning (a.k.a. themes) within the qualitative data sets. The qualitative data analysis was analysed by free listing, and then subcategorised based on emergent themes.

Results

For the purpose of this paper we have narrowed down our findings to those relevant to schools within the two blocks highlighting activities and facilities provided by them and their role as change agents.

Across the nine study villages, information was gathered from 67 mothers, 58 paternal grandmothers using FDGs and 49 key respondents in the KIIs.

For peer review only - http://bmjopen.bmj.com/site/about/guidelines.xhtml

Table 1. Summary of interviewers (numbers by participant group and type of interaction) across the study villages of Ghatol and Kushalgarh

Participant group	*Type of interaction (FGD/KII)*	*N (Ghatol – 5 study villages)*	*N (Kushalgarh – 4 study villages)*	*N (total across the 9 study villages)*
ANM	KII	4	3	7
ASHA	KII	7	6	13
AWW	KII	7	6	13
School Teacher	KII	5	4	9
Sarpanch/Ward Panch	KII	4	3	7
Mothers	FDG	36	31	67
Grandmothers	FDG	30	28	58

Social mapping and transect walk exercises were also conducted in each of the nine study villages to map Water, Sanitation and Hygiene (WASH) as well as energy resources. Two overarching themes were identified during interviews and group discussions: the potential of schools as a method for community mobilization and education at a household and community level.

Figure 2. Connecting subthemes informing Theme 1: Schools as a method for community mobilisation and education at a household level.

Enrolment

Evidence from the interviews showed that school enrolment was conducted by the means of door to door identification by the primary school teachers. All children above five years of age were identified and validated with birth listing done by FHWs. The teachers across the nine villages reported this method to be successful, allowing a recruitment rate of 100%, with no children under 5 years not attending school.

"There are no children who are not in school. All children above 5 years come to school."
[School Teacher, Kushalgarh]

For peer review only

For peer review only - http://bmjopen.bmj.com/site/about/guidelines.xhtml

In Ghatol, it was reported that some children did not attend governmental school given the existence of a private school in the vicinity of three out of five study villages. This was, however, not the case for Kushalgarh where none of the study villages had access to private schools and all enrolled students attended government schools.

"School enrolment is not a problem in this village. Once a year we do a door to door survey to identify school going children. Some families who can afford to send their children to the private do so, rest send them to the government school." [School Teacher, Ghatol]

High school enrolment levels across the nine villages studied indicate the potential reliability of schools as an intervention delivery platform. Furthermore, the regularity with which children were found to attend school and the degree of parental participation in school activities are important considerations.

School attendance

Teachers and mothers reported school attendance to be high throughout the year. Absenteeism was identified as a problem mainly during harvesting season by teachers in both blocks, specifically in Ghatol where school-aged girls were kept at home to take care of younger siblings whilst boys assisted the family in the agricultural work.

"Children mostly miss school when there is harvesting season. Sometimes the entire family gets busy so there is no one to send the child to school. The girls also miss school when they have to take care of their little siblings, but rarely." [School Teacher, Ghatol]

In cases where students were absent for long stretches of time, it was found that teachers conducted home visits to explore the reason for the non-attendance, hence playing an important role as change agents.

Parental participation in schools

Across a majority of schools studied, parent-teacher interaction was reported to be positive, but not optimal. Parents generally reported attending two kinds of school events; (1) National day celebrations in school (e.g. Independence Day or Republic Day) or (2) if a parent was summoned to discuss their child's progress. School Management Committee (SMC) meetings were also held in the school once per month. Both teachers and mothers, from the study village,were of the opinion that parents rarely

For peer review only - http://bmjopen.bmj.com/site/about/guidelines.xhtml

visited schools. Besides formal school meetings, some teachers reported organizing meetings at the community level. Whilst not offered at all schools, the teachers conducting such meetings felt that they were able to effectively engage with the community. However, it was difficult to maintain the regularity of such events due to teacher shortages.

"We go inside the village to interact with the parents. The School Management Committee helps to fix a date, time and place for these meetings. These meetings are useful. [...] It is easy for them to attend those meetings, but we cannot do it frequently." [School Teacher, Ghatol]

Parental participation in homework

According to the teachers from both blocks, homework was given regularly to reinforce the lessons taught. The response to and success in completing the tasks correctly, however, varied across children in the two study blocks and can broadly be divided into three categories: 1) Children who failed to complete the tasks entirely, 2) Children who completed the tasks, but with some errors and 3) Children who completed the tasks with no errors. The teachers mentioned in the interviews that they believe this difference in performance to be at least partially attributable to the level of engagement of the children's family members.

"Only educated mothers can help. We just tell the child to study." [Mothers, Ghatol and Kushalgarh]

The first category of students was mostly those who stayed with their grandparents or the parents who were illiterate and therefore had little to no support for in their education.

"Most children stay with the grandparents. They do not have any environment for studying at home. Homework is done at schools." [School Teacher, Kushalgarh]

The second category of students were those where the child's mother was educated till 5th standard or had a school going elder sibling who assisted him/her. Occasionally, the parents of children in the third category were engaged in teaching the child, hence leading to better performance in homework tasks. According to teachers in Kushalgarh, the majority of students belonged to the first category and the remaining few were in the second category. In case of Ghatol, the first category of students was relatively smaller. In general, these results suggest that teachers are important reference point for students and play an essential role in supporting students, especially where limited support is available at home.

For peer review only

Figure 3. Connecting subthemes informing Theme 2: Schools as a method for community mobilisation and education at a community level.

Availability of playgrounds and other facilities/ Games

The presence of playgrounds or outdoor activity areas was noted amongst all except two schools visited. Additionally, physical education teachers and sporting facilities were available in a few schools. It was reported that facilities were mostly only operational during summer rather than rainy season. In the KIIs, several teachers noted they incorporate play activities in the last period of every school day, since no such opportunities were available at the children's homes. The teachers believe this to be essential for the development of the children, promoting both their physical and mental wellbeing.

"Playing is very necessary for the holistic development of children. We devote the last period each day for that." [School Teacher, Ghatol]

FGDs showed that not all parents were convinced of the usefulness of play activities for their children.

"Parents sometime question us why we play games, school is for studying not playing." [School Teachers, Kushalgarh]

This highlights another potential area where teachers could use their positive standing in the local community to influence and change current practices.

Mid-day meals

All teachers, PRI members and mothers responded that the mid–day meal was prepared in the school and served regularly to students. The menu for each day was fixed by the Education Department, with diet plans including cereals such as wheat and rice, pulses, vegetables. Fruit was also served once a week. The food was prepared by a cook and an assistant under the supervision of the principal or other teachers.

"I have seen food being served in schools regularly. Sometimes I go to check the quality also." [Ward Panch, Ghatol]

No concerns regarding school meal quality were raised in the FGDs with mothers, nor in the interviews with PRIs. The teachers, especially from schools in Kushalgarh, were of the opinion that nutrition diversity offered in mid-day meals was superior to foods served to children at their homes.

For peer review only - http://bmjopen.bmj.com/site/about/guidelines.xhtml

"Yes, we provide food to children regularly. [...] The food that they get in schools is better than what they get in home. Dal is not cooked in the homes every day. Fruit is also an attraction among kids."
[School Teachers, Kushalgarh]

Furthermore, it was reported that the general standards of cleanliness and hygiene, e.g. when storing, preparing and serving food, were higher at the schools than those encountered at home: at schools, clean water was always used to prepare and cook meals and hygiene practices were implanted for hands, cooking equipment and cutlery.

Health and WASH

The school adopted different approaches to improve student health and hygiene practices. For health, an annual check-up was conducted each year by ANMs. The teachers explained that ANMs came with adequate medicine for camps, and mostly provided first aid treatment. They also identified children who needed special medical attention. Health services were also available from their local AWC. The teachers felt this was a successful and necessary initiative, given the generally limited knowledge about health and hygiene in the local community.

Hand pumps available in the premises of most schools, were used as a drinking water source though the water quality was not tested regularly – something which potentially could be incorporated in monitoring and evaluation. In a majority of schools, the same hand pumps were also used for handwashing. In a few schools, special filtering plants were present. Toilets were available for children, in schools; while only few had separate facilities for boys and girls. Overall, the toilets in most schools were in an unsatisfactory condition, with a large proportion of toilets dysfunctional.

Several teachers reported the provision of special programmes related to diarrhoea prevention and hygiene promotion in schools. In these programmes, awareness was generated about health and hygiene practices. The teachers felt that children proved to be an effective channel to pass on this information at home, hence fostering better understanding about nutrition, health and hygiene practices, also across generations.

"We tell the students about good hygiene practices like handwashing, taking a bath every day, cutting nails. They then go and tell their family. Spreading messages through children are very effective."
[School Teacher, Ghatol]

For peer review only

Discussion

Appropriate supplementary Infant and Young Child Feeding (IYCF) are inherently linked with growth and development, especially in children younger than 24 months. Efforts to improve IYCF hence remain a potent intervention target. Awareness generation among mothers and families with young children is crucial for successful intervention implementation.

Traditionally, the role of schools is to build human capital and social cohesion. In our study, schools have been shown to be an important and viable setting for community mobilisation in the context of nutrition, health and hygiene practices. The qualitative evidence from the FDGs and KIIs, as well as intervention development and the piloting of the fore-mentioned interventions, indicate that schools may indeed represent a feasible potential platform for community mobilisation. Enrolment rates were high across the nine villages. Schools and associated staff were dedicated to encouraging parent involvement in the school and discouraging student absenteeism. Initiatives such as the mid-day meal, play opportunities and education on health and hygiene (e.g. through the WASH) All highlight the schools' potential for community engagement and reliability as an intervention delivery platform. Furthermore, schools can act as research sites, playing a crucial role in capturing the diversity and dynamics that exist in particular communities whilst acting as the 'beating heart' of villages.

In this context, teachers appeared to be a crucial stakeholder in bringing about positive change in local communities, acting as a reliable and sophisticated source of information regarding the communities they serve alongside being potential change agents for children, their parents and the wider community via; (a) their connections and respect from their local communities and (b) their interest in co-designing interventions with us. Thus, engaging with the school teachers as in further analysis of the problem and co-designing any future interventions holds merit. This has been recognized locally and was translated into intitiatives of the District Collector if Bansawar, including the Swachhta Pakhwada and Alakh programmes.

It is evident from the literature that children have acted as agents of change in multiple health-related community interventions to improve their own and their parents' health [14][15]. Studies have demonstrated that children learn about a health topic and share that information with their parents or their community, thereby fostering an increase in health awareness and health promoting behaviours across generations [16][17]. This, combined with children's quick learning ability and regular access to high-quality information through schools and associated staff, gives children the potential to become positive community influencers. Future studies utilising the PANChSHEEEL model will investigate this further.

For peer review only - http://bmjopen.bmj.com/site/about/guidelines.xhtml

Conclusion

Schools are likely to act as a central theme in deployment for behaviour change interventions, owing to the fact that they form a central part of the child's daily routine in addition to schools being a centre of community activities. These activities could be incorporated into the ongoing programmes, through either leveraging of the mid-day meal program or other such child development schemes that will provide opportunities to use these interactions to improve the wider determinants of healthy living in children as well as their families.

Developing a supportive environment around schools, geared towards reinforcing messaging on healthy practices and using children as change agents to bring about community change therefore requires further investigation.

For peer review only

Ethics approval and consent to participate: The PANChSHEEEL Project protocol, including qualitative and quantitative data collection methods as well as the tools obtained ethical approval from the UCL ethics committee (REF NO: 4032/002) in United Kingdom and the Sigma-IRB (REF NO: 10025/IRB/D/17-18 REF NO: 4032/002) in India.

Consent for publication: Not applicable.

Declaration of Conflicting Interests: The authors declared no potential conflicts of interest with respect to the research, authorship, and/or publication of this article.

Data Sharing Statement: The data of this study is available from the corresponding author upon reasonable request.

Funding: This research was supported by the MRC Global Challenges Research Fund [grant number: MR/P024114/1]. Prof Monica Lakhanpaul was supported by the National Institute for Health Research (NIHR) Collaboration for Leadership in Applied Health Research and Care (CLAHRC) North Thames at Bart's Health NHS Trust. Dr. Priti Parikh is currently supported by the BBOXX/Royal Academy of Engineering Senior Research Fellowship.

Author Contributions: ML, MCL, PP, RK, and SS conceived the original concept of the study and designed the research methodology. SR, ML, PraP, RK, HC, SS, SPP, TS, and PP carried out the interviews, analysed the data and contributed to manuscript writing. ML, LM, PP, SR, HC, NS, SS, SPP, and RK validate the study and revised the manuscript critically for important intellectual content. SA and ICD wrote the manuscript, contributed to the data analysis, edited the final manuscript and prepared for submission. ML had primary responsibility for the final content. All authors read and contributed to reviewing the analysis of the data, the designing of the manuscript, and the approval of the final manuscript.

Acknowledgments: On behalf of the PANChSHEEEL team (Professor Monica Lakhanpaul, Susrita Roy, Dr Lorna Benton, Professor Marie Lall, Rajesh Khanna, Professor Virendra Kumar Vijay, Sanjay Sharma, Dr Logan Manikam, Neha Santwani, Dr Hanimi Reddy, Hemant Chaturvedi, Shereen Allaham, Satya Prakash Pattanaik, Tol Singh, Pramod Pandya, Priyanka Dang, Dr Priti Parikh), we gratefully acknowledge the input from our community champions and field team which made this project possible. We would like to thank our research partners; Madhav Foundation (Ritu Chhabria & Ritu Prakash), EKAM Foundation (Dr.Sai Lakshmi, Ms. Neeta Karal Nair, Mr. Manoharan, Dr. Sathya Jegannathan and Ms. Benita), Foundation for Research in Community Health India (Dr Nerges Mistry & Dr. Shilpa Karvande), Society for Nutrition Education & Health Action (SNEHA; Dr Nayreen Daruwalla and Dr David Osrin) and our technical advisory board (Prof Anita Saxena, Mr Anand Karve, Prof V M Chariar, Prof T. Sundararaman, Dr Ian Warwick, Dr Ramesh Mehta, Prof Atul Singhal, John Pelton, Professor Sachin Maheshwari, Dr Sofia Strummer, Mr Himanshu Parikh & Dr Amita Kashyap) for their input throughout the study. The opinions in this paper solely reflect the views of the authors, not of organisations who financially supported this research. All data used in this research is the property of the authors. It is only obtaineable by email from the corresponding author.

Word count: 4636

For peer review only

References

1. Mitra M, Kumar PV, Chakravarty S, Bharati P. Nutritional status of Kamar tribal children in Chhattisgarh. Indian J Pediatric 2007, 74(4); 381-4.

2. Imdad A, Yakoob MY, Bhutta ZA. Impact of maternal education about complementary feeding and provision of complementary food on child growth in developing countries. BMC Public Health 2011; 11, Suppl 3.

3. World Health Organisation. Infant and Young Child Feeding: Model Chapter for Textbooks for Medical Students and Allied Health Professionals. SESSION 1: The importance of infant and young child feeding and recommended practices. Geneva, 2009. Available from: https://www.ncbi.nlm.nih.gov/books/NBK148967/

4. Chakrabarti S, Raghunathan K, Alderman H, Menon P, Nguyen P. India's Integrated Child Development Services programme; equity and extent of coverage in 2006 and 2016. *Bull World Health Organ*. 2019;97(4):270-282. doi:10.2471/BLT.18.221135

5. World Health Organisation (WHO). Ottawa Charter for Health Promotion. Ottawa, 1986. Available at: http://www.euro.who.int/__data/assets/pdf_file/0004/129532/Ottawa_Charter.pdf?ua=1 [Accessed: 10/09/20]

6. Herlitz L, MacIntyre H, Osborn T et al. The sustainability of public health interventions in schools: a systematic review. Implementation Sci 15, 4 (2020). Available at: https://doi.org/10.1186/s13012-019-0961-8 [Accessed: 08/09/20]

7. Mukamana O, Johri M. What is known about school-based interventions for health promotion and their impact in developing countries? A scoping review of the literature. Health Education Research, Volume 31, Issue 5, October 2016, Pages 587–60. Available at: https://doi.org/10.1093/her/cyw040 [Accessed: 12/09/20]

8. Bresee S, Caruso BA, Sales J, Lupele J, Freeman MC. 'A child is also a teacher': exploring the potential for children as change agents in the context of a school-based WASH intervention in rural Eastern Zambia. *Health Education Research*, Volume 31, Issue 4, August 2016, Pages 521–534.

9. Brown T, Summerbell C. Systematic review of school-based interventions that focus on changing dietary intake and physical activity levels to prevent childhood obesity: an update to the obesity guidance produced by the National Institute for Health and Clinical Excellence. Obes Rev 2009;

For peer review only - http://bmjopen.bmj.com/site/about/guidelines.xhtml

10. Khambalia AZ, Dickinson S, Hardy LL, Gill T, Baur L. A synthesis of existing systematic reviews and meta-analyses of school-based behavioural interventions for controlling and preventing obesity. Obes Rev 2012; 13:214–33.

11. Li M, Li S, Baur LA, Huxley RR. A systematic review of school-based intervention studies for the prevention or reduction of excess weight among Chinese children and adolescents. Obes Rev 2008; 9:548–59.

12. Ribeiro IC, Parra DC, Hoehner CM, Soares J, Torres A, Pratt M, Legetic BD, Malta DC, Matsudo VR, Ramos LR, et al. School-based physical education programs: evidence-based physical activity interventions for youth in Latin America. Glob Health Promot 2010; 17:5 –15.

13. Verstraeten R, Roberfroid D, Lachat C, Leroy JL, Holdsworth M, Maes L, Kolsteren PW. Effectiveness of preventive school-based obesity interventions in low- and middle-income countries: a systematic review. Am J Clin Nutr. 2012 Aug;96(2):415-38. doi: 10.3945/ajcn.112.035378. Epub 2012 Jul 3. PMID: 22760565.

14. Onyango-Ouma, W. (2003). Children as partners in health communication in a Kenyan community. Anthropology in Action, 10(1), 25-33.

15. Sedighi I, Nouri S, Sadrosadat T, Nemati R, Shahbazi, M. Can children enhance their family's health knowledge? An infectious disease prevention program. Iran J Pediatr, 22(4), 493-498.

16. Gadhoke P, Christiansen K, Rowan M, Aniliker J, Frick K, Gittelsohn, J. Formative research supports a change agent approach to prevent obesity in American Indian households. The FASEB Journal, 26(1). 2012. doi: 10.1096/fasebj.26.1_supplement.389.7

17. Mosavel M, Genderson MW. Daughter-initiated cancer screening appeals to mothers. J Cancer Educ, 31(4), 767-775. 2016. doi:10.1007/s13187-015-0949-2

For peer review only

Figure 1. Inclusion and exclusion criteria for the study villages and schools

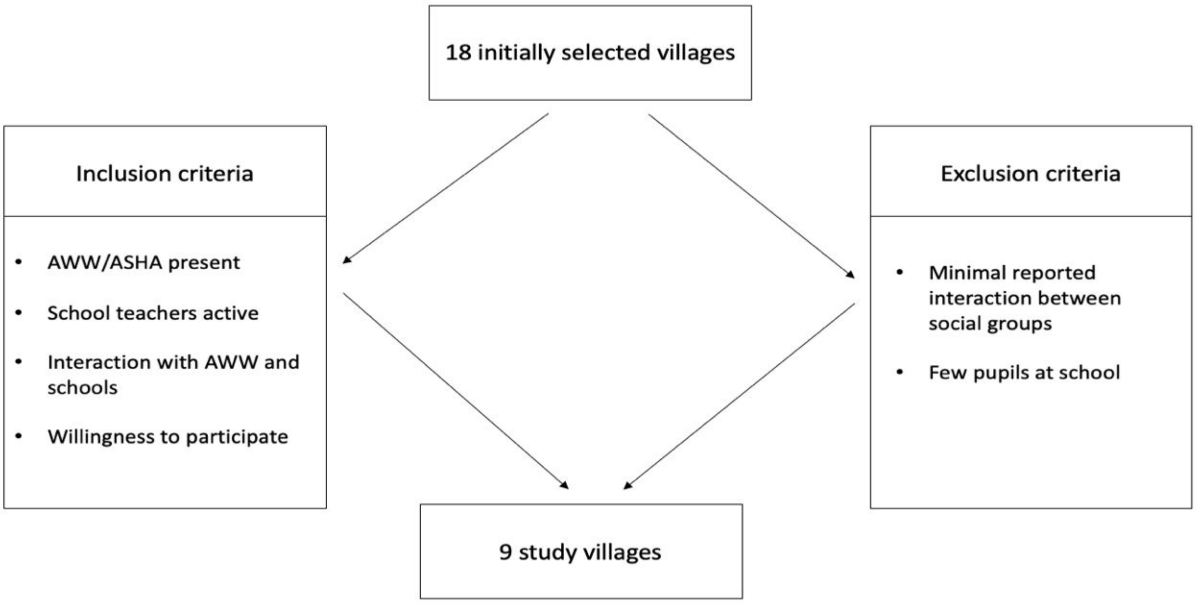

Figure 2. Connecting subthemes informing Theme 1: Schools as a method for community mobilisation and education at a household level.

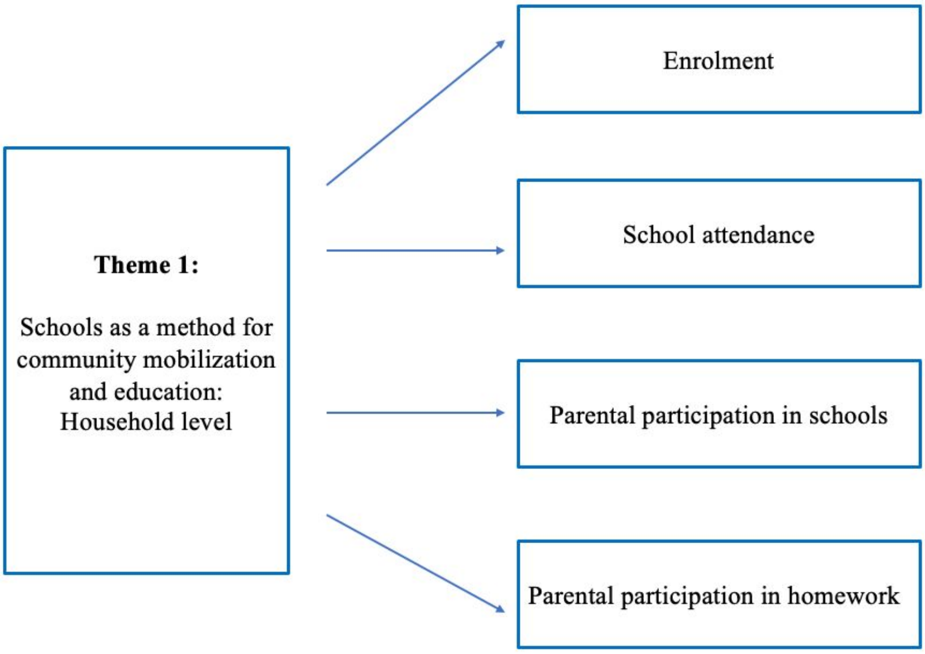

For peer review only - http://bmjopen.bmj.com/site/about/guidelines.xhtml

Figure 3. Connecting subthemes informing Theme 2: Schools as a method for community mobilisation and education at a community level.

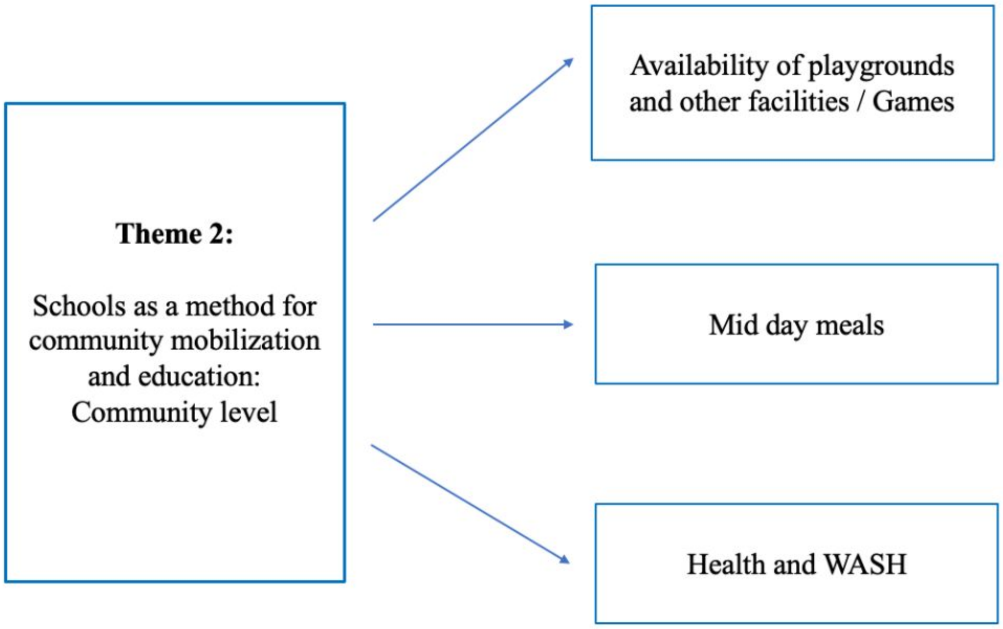

eview only

Manuscript: Title: The Role of Schools in the PANChSHEEEL Project – an Approach to Identify and Address Factors Influencing Infant and Young Child Feeding Practices and Nutrition in India.

Consolidated criteria for reporting qualitative studies (COREQ): 32-item checklist

Developed from:
Tong A, Sainsbury P, Craig J. Consolidated criteria for reporting qualitative research (COREQ): a 32-item checklist for interviews and focus groups. *International Journal for Quality in Health Care*. 2007. Volume 19, Number 6: pp. 349 – 357

No. Item	Guide questions/description	Reported on Page #
**Domain 1: Research team and reflexivity**		
Personal Characteristics		
1. Interviewer/facilitator	Which author/s conducted the interview or focus group?	Page 7-9
2. Credentials	What were the researcher's credentials? E.g. PhD, MD	Page 7 & 8
3. Occupation	What was their occupation at the time of the study?	Page 7 & 8
4. Gender	Was the researcher male or female?	Page 7 & 8
5. Experience and training	What experience or training did the researcher have?	Page 8
Relationship with participants		
6. Relationship established	Was a relationship established prior to study commencement?	Page 6
7. Participant knowledge of the interviewer	What did the participants know about the researcher? e.g. personal goals, reasons for doing the research	Page 6-8
8. Interviewer characteristics	What characteristics were reported about the interviewer/facilitator? e.g. Bias, assumptions, reasons and interests in the research topic	Page 6

For peer review only

Domain 2: study design		
Theoretical framework		
9. Methodological orientation and Theory	What methodological orientation was stated to underpin the study? e.g. grounded theory, discourse analysis, ethnography, phenomenology, content analysis	Page 2 & 6
Participant selection		
10. Sampling	How were participants selected? e.g. purposive, convenience, consecutive, snowball	Page 8
11. Method of approach	How were participants approached? e.g. face-to-face, telephone, mail, email	Page 6-8
12. Sample size	How many participants were in the study?	Page 7, 9 & 10
13. Non-participation	How many people refused to participate or dropped out? Reasons?	Page 7
Setting		
14. Setting of data collection	Where was the data collected? e.g. home, clinic, workplace	Page 8
15. Presence of non-participants	Was anyone else present besides the participants and researchers?	Page 8
16. Description of sample	What are the important characteristics of the sample? e.g. demographic data, date	Page 6 & 8
Data collection		
17. Interview guide	Were questions, prompts, guides provided by the authors? Was it pilot tested?	Yes, a topic guide was provided. Page 9
18. Repeat interviews	Were repeat interviews carried out? If yes, how many?	N/A
19. Audio/visual recording	Did the research use audio or visual recording to collect the data?	Page 9
20. Field notes	Were field notes made during and/or after the interview or focus group?	Page 9

For peer review only

21. Duration	What was the duration of the interviews or focus group?	N/A.
22. Data saturation	Was data saturation discussed?	Page 8 & 9
23. Transcripts returned	Were transcripts returned to participants for comment and/or correction?	Page 9
**Domain 3: analysis and findings**		
Data analysis		
24. Number of data coders	How many data coders coded the data?	Page 9
25. Description of the coding tree	Did authors provide a description of the coding tree?	No.
26. Derivation of themes	Were themes identified in advance or derived from the data?	Derived from the data. Page 10-14
27. Software	What software, if applicable, was used to manage the data?	Page 9
28. Participant checking	Did participants provide feedback on the findings?	Page 9
Reporting		
29. Quotations presented	Were participant quotations presented to illustrate the themes/findings? Was each quotation identified? e.g. participant number	Yes.<br>Page 10-14
30. Data and findings consistent	Was there consistency between the data presented and the findings?	Yes.<br>Page 10-14
31. Clarity of major themes	Were major themes clearly presented in the findings?	Yes.<br>Page 10 & 13
32. Clarity of minor themes	Is there a description of diverse cases or discussion of minor themes?	Yes<br>Page 10-14

For peer review only

BMJ Open

The Role of Schools in community mobilisation to improve IYCF practices in 6-24 month old Tribal children in the Banswara district, India – Findings from the qualitative PANChSHEEEL study

Journal:	*BMJ Open*
Manuscript ID	bmjopen-2020-047741.R1
Article Type:	Original research
Date Submitted by the Author:	01-Nov-2021
Complete List of Authors:	Lakhanpaul, Monica; University College London, Institute of Child Health<br>Roy, Susrita; Save The Children<br>Lall, Marie; University College London, Institute of Education<br>Chaturvedi, Hemant; Save the Children<br>Khanna, Rajesh; Save the Children<br>Allaham, Shereen ; University College London Institute of Epidemiology and Health Care, Department of Epidemiology and Public Health; Aceso Global Health Consultants Ltd<br>Demel, Isabel-Cathérine; King's College London Faculty of Life Sciences and Medicine, Guy's, King's and St.Thomas' School of Medicine<br>Benton, Lorna; University College London Institute of Child Health, Population, Policy and Practice<br>Vijay, Virendra ; Indian Institute of Technology Delhi<br>Sharma, Sanjay ; University College London, Engineering for International Development Centre<br>Manikam, Logan; University College London Institute of Epidemiology and Health Care, Department of Epidemiology and Public Health<br>Santwani, Neha; Save the Children<br>Pattanaik, Satya Prakash ; Save the Children<br>Singh, Tol; Save the Children<br>Pandya, Pramod; Save the Children<br>Dang, Priyanka; Save the Children<br>Parikh, Priti; University College London, CEGE
**Primary Subject Heading**:	Public health
Secondary Subject Heading:	Global health, Nutrition and metabolism, Paediatrics, Qualitative research, Health services research
Keywords:	PUBLIC HEALTH, Community child health < PAEDIATRICS, NUTRITION & DIETETICS, International health services < HEALTH SERVICES ADMINISTRATION & MANAGEMENT

SCHOLARONE™
Manuscripts

I, the Submitting Author has the right to grant and does grant on behalf of all authors of the Work (as defined in the below author licence), an exclusive licence and/or a non-exclusive licence for contributions from authors who are: i) UK Crown employees; ii) where BMJ has agreed a CC-BY licence shall apply, and/or iii) in accordance with the terms applicable for US Federal Government officers or employees acting as part of their official duties; on a worldwide, perpetual, irrevocable, royalty-free basis to BMJ Publishing Group Ltd ("BMJ") its licensees and where the relevant Journal is co-owned by BMJ to the co-owners of the Journal, to publish the Work in this journal and any other BMJ products and to exploit all rights, as set out in our licence.

The Submitting Author accepts and understands that any supply made under these terms is made by BMJ to the Submitting Author unless you are acting as an employee on behalf of your employer or a postgraduate student of an affiliated institution which is paying any applicable article publishing charge ("APC") for Open Access articles. Where the Submitting Author wishes to make the Work available on an Open Access basis (and intends to pay the relevant APC), the terms of reuse of such Open Access shall be governed by a Creative Commons licence – details of these licences and which Creative Commons licence will apply to this Work are set out in our licence referred to above.

Other than as permitted in any relevant BMJ Author's Self Archiving Policies, I confirm this Work has not been accepted for publication elsewhere, is not being considered for publication elsewhere and does not duplicate material already published. I confirm all authors consent to publication of this Work and authorise the granting of this licence.

For peer review only

The Role of Schools in community mobilisation to improve IYCF practices in 6-24 month old Tribal children in the Banswara district, India – Findings from the qualitative PANChSHEEEL study

Monica Lakhanpaul[1,2]
Susrita Roy[3]
Marie-Carine Lall[4]
Hemant Chaturvedi[5]
Rajesh Khanna[3]
Shereen Allaham[6,7]
Isabel-Catherine Demel[7,8]
Lorna Benton[9]
Virendra Kumar Vijay[10]
Sanjay Sharma[11]
Logan Manikam[6,7]
Neha Santwani[3]
Satya Prakash Pattanaik[5]
Tol Singh[5]
Pramod Pandya [5]
Priyanka Dang[3]
Priti Parikh[11]

Institutions

1. Population, Policy and Practice, UCL Great Ormond Street Institute of Child Health, 30 Guildford Street, London WC1N 1EH, UK
2. Whittington Health NHS Trust, London, UK, N19 5NF
3. Save the Children, National Support Office, Gurugram, India
4. Institute of Education, University College London, UK
5. Save the Children, Rajasthan, India
6. Department of Epidemiology and Public Health, University College London Institute of Epidemiology and Health Care, London, UK.
7. Aceso Global Health Consultants Limited, London, UK
8. Department of Life Sciences & Medicine, King's College London GKT School of Medical Education, London, UK
9. Great Ormond Street Institute of Child Health, University College of London, UK.
10. Indian Institute of Technology (IIT) Delhi, India
11. Engineering for International Development Centre, University College London, London WC1E 7HB

Corresponding Author:
Monica Lakhanpaul
Population, Policy and Practice, UCL Great Ormond Street Institute of Child Health, 30 Guildford Street, London WC1N 1EH, UK
Email: m.lakhanpaul@ucl.ac.uk

Word count: 5706

For peer review only - http://bmjopen.bmj.com/site/about/guidelines.xhtml

ABSTRACT

Objective: India has been struggling with infant malnutrition for decades. There is a need to identify suitable platforms for community engagement to promote locally feasible, resource efficient Infant and Young Child Feeding (IYCF) interventions. This study aims to explore if and how schools could represent a site for community engagement in rural India, acting as innovation hubs to foster positive change in partnership with the Angawadi centres(AWCs).

Design: 5-phase formative study; A parallel mixed methods approach structured by a socio-ecological framework was used for data collection at individual, household and community levels. This paper focuses on the qualitative findings.

Setting: This study was undertaken in nine villages within two blocks, 'Ghatol' and 'Kushalgarh', in the Banswara district of Rajasthan, India.

Participants: 17 schools were identified. Interviews were conducted with local opinion leaders and representatives in the education sector, including principals, schoolteachers, Block and District Education officers. Across the 9 study villages, information was gathered from 67 mothers, 58 paternal grandmothers using FDGs and 49 key respondents in KIIs.

Results: Schools were considered an important community resource. Challenges included limited parental participation and student absenteeism; however, several drivers and opportunities were identified which may render schools a suitable intervention delivery site. Enrolment rates were high, with schools and associated staff encouraging parental involvement and student attendance. Existing initiatives, including the mid-day meal, play opportunities and education on health and hygiene, further highlight the potential reliability of schools as a platform for community mobilisation.

Conclusions: Schools have been shown to be functional platforms frequently visited and trusted by community members. With teachers and children as change agents, schools could represent a suitable setting for community mobilisation in future wider scale intervention studies. Expanding the supportive environment around schools will be essential to reinforce healthy IYCF practices in the long term.

Abstract word count: 298 words

KEYWORDS: Global Health, Nutrition, Community Child Health, Complementary Feeding, IYCF Practices, School-Based Interventions, Public Health, Angawadi

For peer review only - http://bmjopen.bmj.com/site/about/guidelines.xhtml

Article Summary

Strengths and Limitations of this study

Strengths

- The PANChSHEEEL (Participatory Approach for Nutrition in Children: Strengthening Health Education Engineering and Environment Linkages - HEEE) project was an interdisciplinary study, designed to explore an array of important interconnected themes such as health, education, engineering and environment that influence Infant and Young Child Feeding (IYCF) practices and nutrition in India with the aim of developing a socio-culturally appropriate, tailored, innovative and integrated multisectoral HEEE Nutrition package to optimise IYCF practices for children aged 6-24 months.
- This study established the advantages of using schools to bring communities together and disseminate information regarding IYCF practices whilst exploring the potential of working with children as community change agents.

Limitations

- Due to time and resource constraints, the study relied on the coverage and completeness of external data, e.g. a list of the children under the age of 2 years resident in the study area (prepared by Anganwadi Worker and/or Accredited Social Health Activist), as a starting point for the data collection.
- The study covered only 9 villages in total across two blocks in the state of Rajasthan. Despite the main formative study being led across 18 villages, the phase of study directly related to testing the feasibility and acceptability of selected pilot school-based interventions were only conduced in 9 villages.
- The data collection phase of this study fell into the months of January to March, the peak of agricultural season in the Banswara district. This impacted our sample size due to absence of functionaries at schools for KIIs and FDGs and/or participants in our household survey.

For peer review only

Introduction

Many low-and-middle-income countries (LMIC) have been grappling with the problem of malnutrition for decades. Given its persistent and chronic nature, malnutrition affects a large proportion of the population in these countries. The triple burden of malnutrition presents itself in the form of stunting, wasting and anaemia - all of which are especially common in women and children.

It is well known that the period between 6 and 24 months of age represents a critical time for infant growth. [1] In LMIC, stunting is generally the consequence of infectious disease and low nutrient intake, particularly inadequate energy and protein intake, relative to nutritional requirements [2]. In addition to infectious disease prevention strategies, supplementary Infant and Young Child Feeding (IYCF) interventions targeting this critical period are most effective in reducing malnutrition and promoting childhood growth and development [1].

In response to this, in 1975 India introduced an 'Integrated Child Development Service' (ICDS), – an intervention initiative including supplementary nutrition, immunisation and pre-school education to enhance health outcomes for pregnant women, breastfeeding mothers and children up to age 6. [3] The National Programme of Nutritional Support linked to Primary Education (Midday Meal Initiative) delivering hot cooked lunches to all the nation's school children is one of ICDS's flagship interventions, along with the introduction of rural child care centres, so called AWC. Linked with schools, the AWCs are aimed at providing services and skilled staff to enhance nutritional outcomes, basic medical aid and preschool education for 2-5 year olds and their mothers in the community setting. Whilst this has led to a significant improvement in the care continuum from pregnancy to early childhood, households from the lowest quintiles of the economic strata and women with poor schooling levels remain difficult to reach. [2] Identifying occasions and settings where community members gather at regular intervals with children will hence be crucial to increase the reach of similar interventions going forward, especially in rural states.

Schools appear to be one potential setting for community mobilisation and implementation of interventions, as they offer continuous, intensive contact with children. By contributing to students' health literacy and behaviours, school-based health promotion can be particularly valuable in the context of developing countries where low public health literacy and high burden of disease are faced. [4] Furthermore, children have been demonstrated to be change agents who are able to transfer information and play as an active-partner in the school-home relation [5]. In a literature review on school-based health promotion and their impact in developing countries, it was found that a majority of school-based interventions addressed nutrition, hygiene, oral health and communicable diseases [4],

For peer review only - http://bmjopen.bmj.com/site/about/guidelines.xhtml

including nutrition education and hygiene awareness campaigns to reduce the incidence of worm infections. Implementation was carried out by schoolteachers, nurses and other community members, with study results suggesting an improvement in the knowledge and attitudes of their target population[4,5].

In an attempt to maximise the potential of schools and achieve greater convergence, and an improved continuum of care for early childhood, the Government of India subsequently scaled up the so-called 'Rajasthan model'– an initiative that lead to the establishment and co-location of 11,000 Anganwadi Centres (AWCs) with schools by mid-2017 in rural India. [6]

This soon led to schools emerging as a logical platform for intervention delivery in the context of the PANChSHEEEL project (Participatory Approach for Nutrition in Children: Strengthening Health Education Engineering and Environment Linkages). At the conceptualisation stage, schools, in conjunction with the government-funded, co-located AWCs, were thus considered as a potential platform and resource for community mobilisation to improve IYCF practices in rural India.

In this paper, we present the qualitative findings from our formative study, exploring the acceptability and feasibility of schools as a platform for community mobilisation and health promotion activities related to IYCF practices and the role of children as community change agents.

Methodology

Study design

This present study forms part of a wider project: The PANChSHEEEL (Participatory Approach for Nutrition in Children: Strengthening Health Education Engineering and Environment Linkages) - initiative; a project involving a 5-phase formative interdisciplinary study, designed to explore health, education and environmental (HEEE) factors that influence IYCF practices and nutrition in India. Informed by the socioecological model, the study aimed to develop a socio-culturally appropriate, tailored, innovative and integrated cross sector HEEE Nutrition package to support optimal IYCF practices for children in rural India aged 6-24 months. [7] [8] [9]

In this project, members from University College London (UCL), Institute of Child Health, Institute of Education and Faculty of Engineering Sciences, and expertise from; Save the Children- India, Indian Institute of Technology, New Delhi (IIT-Delhi) and Jawaharlal Nehru University, New Delhi (JNU) formed the CT. The participatory approach actively involved local Community Researchers (CR), Community Champions (CC), and Community Members (CM) selected from the nine villages within Ghatol and Kushalgarh Block of Banswara district of Rajasthan (India).

For peer review only - http://bmjopen.bmj.com/site/about/guidelines.xhtml

The study was conducted in tribal areas including households from the lowest quintiles of the economic strata and women with poor schooling levels. At the outset of the study, understanding and reflecting on the village structure and positioning of schools in relation to other village resources was key for the field team. Village profiles were therefore created by the means of social mapping and community profiling. Social mapping was conducted using a participatory approach, with 10-15 participants per village being identified by key figures in the community (e.g. elected village representative, school teachers and other local leaders). Field investigators acted as facilitators, explaining the purpose of drawing a village map and working with the participants to produce it. The first step in this process was drawing the concrete or pucca roads, followed by the foot-roads or kuccha roads. Important institutions (e.g. temples, government schools), houses and water sources (e.g. hand pumps, canals) were then marked on the map. Participants were required to discuss with one another before reaching a consensus on the final map. The participants' map was then validated by the village representatives. [9]

Phase 1 of the study focused on identifying and documenting local practices with regard to IYCF practices, sanitation, education & access to local resources (such as energy and water). The existing Government of India (GOI) initiatives in the study sites were also mapped during Phase 1. Phase 2 involved extensive fieldwork to identify local challenges, drivers, resources, opportunities and needs for children in the first 6-24 months of life at individual, household, community and environmental level. Phases 3, 4 and 5 focused on the analysis and mapping of HEEE linkages, the co-creation and piloting of an IYCF intervention package (IP) using an iterative process, as well as review and dissemination phases of this project.[10] This present paper focuses only on the qualitative findings relating to the potential role of schools in community mobilisation to support improvements in IYCF from the formative phase (Phase 1) . The full study process is presented in two available reports. [11]

Schools selection

The formative research phase commenced after a systematic selection of nine study villages from the two blocks of Banswara, namely Ghatol and Kushalgarh. Out of the 18 villages proposed initially by 'Save the Children India', 9 were selected purposively based on an interaction of the core research team (CT) with 1) the local community, 2) the teachers at the local elementary schools and 3) the Anganwadi workers (AWW) and Accredited Social Health Activist (ASHA) in each village. Schools where the relationship with the local village and functionality of the school itself were poor, e.g. low number of teachers and students, insufficient engagement with the study, were excluded. Secondly, villages where a lack of interaction between different social groups or lack of interest in the project was noted, were subject to exclusion. Finally, the linkage of the schools with the AWCs, e.g. the government-funded centres that provide facilities such as food, vaccines, medical treatments to 2-5-

For peer review only - http://bmjopen.bmj.com/site/about/guidelines.xhtml

year olds alongside assisting mothers in accessing other government schemes/services, was a key determining factor. Schools, where the Anganwadi Centre (AWC) was not directly associated with the school or where the AWW displayed insufficient interaction, were excluded. A summary of the inclusion and exclusion criteria for the study villages and schools is outlined in Figure 1. Overall, 17 schools were identified in the nine selected villages.

Figure 1. Inclusion and exclusion criteria for the study villages and schools

Patients and Public involvement and Engagement

Patient/Participant and Public Involvement and Engagement (PPIE) representatives were identified, selected and engaged in every step of the study from protocol development over study design to the dissemination of the results.

Community members from the nine villages within Ghatol and Kushalgarh Blocks of the Banswara district of Rajasthan were actively supporting the Core Research Team (CT) in the context of PANChSHEEEL's participatory approach. To help steer the project across its phases locally, Community Researchers (CR) from the study blocks Ghatol and Kushalgarh were recruited. The inclusion criteria encompassed (1) being educated, (2) having previous field experience in Banswara district, e.g. understanding the village context and social networks locally, and (3) being conversant in both 'Hindi' and 'Wagdi' languages (given that the most prevalent language dialect in the nine study villages is 'Wagdi'). With the help from the 'Save the Children' Team from the Rajasthan State Office, two suitable individuals were identified. The CR were initially male adult members of the study population who understood the local village and social context, providing special insight about their communities and environment to the study. Both had shown satisfactory engagement in a previous project by 'Save the Children', were local to the study blocks of Ghatol and Kushalgarh, and hence recruited on contractual basis during Phase 1 of the study to support community engagement and data collection. Their main role was to identify potential candidates who could engage actively in project activities, as well as engaging with the Community Champions (CCs), who in turn helped establish linkages with the Community Members (CMs) and improve networks between schools, FLWs and villages.

Aligning with the principle of reflexivity in research, i.e. the continuous process of examining relationships, the dynamics of that relationship and its impact [11], two female researchers were subsequently recruited in addition (for short term). This decision was made in response to the situational dynamics in our study setting [11] and feedback from local community members (such as

For peer review only - http://bmjopen.bmj.com/site/about/guidelines.xhtml

teachers and AWW,) who felt that the female respondents would discuss sensitive topics of breastfeeding and complementary feeding more freely with a female, rather than male, CR in the quantitative household survey. The following criteria were hence set for recruitment purposes: the chosen female community researcher should (1) be a graduate; (2) have experience in interviewing female community members 3) be familiar with the local dialect, i.e. Wagdi and 4) be prepared to travel / commute within the study district.

Community Champions(CCs) were associated voluntarily in the research. They played no direct part in data collection, but did have an important role in being advocates for the study and facilitating access to the community themselves.

Data collection

For the whole PANChSHEEEL study, a mixed-methods approach structured by a socio-ecological framework was employed for data collection. Primary data was collected at an individual, household and community level by the CT and CR together. Qualitative data was obtained using two methods: 1) Key Informant Interviews (KII) with AWWs, ASHAs, ANMs, Elected Panchayat Representatives (PRI members), school teachers and block education officers and 2) Focus Group Discussions (FGD) with mothers and grandmothers of children 0-2 years of age. Whilst not directly involved in the data analysis, findings were presented to CR's at each stage for verification and interpretation purposes.

Participants were asked for consent in the context of both the qualitative and quantitative data collection. Written consent was obtained from participants of the KIIs. Verbal consent was obtained from all participants of FDGs and recorded by the means of an audio device. All interviews were documented with a voice recorder for subsequent analysis. Field notes were taken during the interviews with stakeholders. The coding was carried out by the field team and was done firstly in Hindi, and subsequently, in English.

The members of the research team conducting the qualitative data collection received a two-day training by an experienced co-investigator in the Banswara district in December 2017. This training was followed by a two-day field practice. Data was collected by the CRs with assistance from CCs for scheduling meetings with stakeholders. The CR were supported by the CT and received thorough training on techniques and tools to FGDs, KII and Broad Narrative Group Discussions. PowerPoint Presentations and handouts were used for training and reflective sessions were organised with the CT.

For peer review only - http://bmjopen.bmj.com/site/about/guidelines.xhtml

During the formative phase and stakeholder mapping workshop, relevant stakeholders were identified and recruited. Considering the possibility of snowballing, a sampling method where existing participants inform the recruitment of future participants from among their acquaintances and relevant to the study, the research team was open to any new stakeholder(s) identified during the data collection.

The community members were interviewed in focus group discussions (FGD's) whereas key informants (KII) were interviewed individually. A focus was placed on interviewing paternal grandmothers, primarily due to their significant influence on mothers: after the delivery, it is common for mother and infant to spend the initial postpartum and puerperal period in her husband's living environment. This makes paternal grandmothers key in guiding the mother after the delivery on breast- and complementary feeding practices. All participants in the mothers' group had children below the age of two years; most of them also had an older school-going child. Similarly, all grandmothers had a grandchild under two years of age Interviews were also held with other key informants, namely elected representatives from the village such as Female Health Workers (FHWs), Auxiliary nurse Midwife (ANM), Anganwadi Workers (AWW) and Accredited Social Health Activists (ASHA), Block Education officers, Ward Panchayats and the village school teachers and principals. The recruitment of participants was conducted based on availability and willingness to participate.

Topic Guide

The CT and CR designed thematic guides designed through literature review, expert and stakeholder consultation and evolved through pilot testing with different population sub-sets.

For peer review only - http://bmjopen.bmj.com/site/about/guidelines.xhtml

Data management and analyses

Qualitative data was collected in Hindi or Wagdi and transcribed and translated by local translators who were proficient in Wagdi and Hindi. Research team members who were proficient in Hindi and English translated the Hindi texts to English. At each stage, quality assurance measures were adopted by checking for reverse translation. Coding was done by two junior researchers who had considerable experience of the study sites and contexts. Final consensus was arrived at collectively by the senior researchers of the team. Data was collected from schools in all the nine study villages.

The qualitative data collected was transferred from the recording device to the encrypted laptop of the SCI Nodal Person. The research team also met at the end of each day to share the key observations and reflections about the data based on the notes taken during the KII and FGD.

Audio-recorded data was translated and transcribed alongside detailed summary of field notes prepared. Data from open-ended questionnaire questions were evaluated, discussed and summarised across the various IYCF themes for each intervention point. The coding was conducted by the field team and was done firstly in Hindi, and subsequently, in English.

The initial data collected by CR further underwent respondent validation. Respondent validation was conducted in a workshop involving CCs facilitated by CR under the guidance of 'Save the Children' staff. The steps of a thematic analysis were employed; this means identifying, analysing and interpreting patterns of meaning (a.k.a. themes) within the qualitative data sets. [12] The qualitative data analysis was analysed by free listing, and then subcategorised based on emergent themes.

Results

For the purpose of this paper, we have narrowed down our findings to those relevant to schools within the two blocks.

Across the nine study villages, information was gathered from 67 mothers, 58 paternal grandmothers using FDGs and 49 key respondents in the KIIs. Table 1 illustrates a summary of the interviewers across the study villages of Ghatol and Kushalgarh.

For peer review only - http://bmjopen.bmj.com/site/about/guidelines.xhtml

Participant group	Type of interaction (FGD/KII)	N (Ghatol – 5 study villages)	N (Kushalgarh – 4 study villages)	N (total across the 9 study villages)
ANM	KII	4	3	7
ASHA	KII	7	6	13
AWW	KII	7	6	13
School Teacher	KII	5	4	9
Sarpanch/Ward Panch	KII	4	3	7
Mothers	FDG	36	31	67
Grandmothers	FDG	30	28	58

Table 1. Summary of interviewers (numbers by participant group and type of interaction) across the study villages of Ghatol and Kushalgarh

Findings from both KIIs and FDGs revealed several activities and facilities provided by schools that may render them a suitable site for community mobilisation. The challenges, drivers and opportunities of schools as change agents across the nine examined study villages are summarised in Figure 2.

Figure 2. Overarching themes identified during the thematic analysis of KIIs and FDGs: challenges, drivers, and opportunities.

Challenges

Parental participation in schools

Across a majority of schools studied, parent-teacher interaction was reported to be positive, but not optimal. Parents generally reported attending two kinds of school events; (1) National day celebrations in school (e.g. Independence Day or Republic Day) or (2) if a parent was summoned to discuss their child's progress. School Management Committee (SMC) meetings were also held in the school once per month. Both teachers and mothers, from the study village were of the opinion that parents usually visited formal school meetings and some teachers reported organizing meetings at the community level. Whilst not offered at all schools, the teachers conducting such community meetings felt that they were able to effectively engage with the community. However, it was difficult to maintain the regularity of such events due to teacher shortages.

"We go inside the village to interact with the parents. The School Management Committee helps to fix a date, time and place for these meetings. These meetings are useful. [...] It is easy for them to attend those meetings, but we cannot do it frequently." [School Teacher, Ghatol]

For peer review only - http://bmjopen.bmj.com/site/about/guidelines.xhtml

School attendance

Teachers and mothers reported school attendance to be high throughout the year. Absenteeism was identified as a problem mainly during harvesting season by teachers in both blocks, specifically in Ghatol where school-aged girls were kept at home to take care of younger siblings whilst boys assisted the family in the agricultural work.

"Children mostly miss school when there is harvesting season. Sometimes the entire family gets busy so there is no one to send the child to school. The girls also miss school when they have to take care of their little siblings, but rarely." [School Teacher, Ghatol]

In cases where students were absent for long stretches of time, it was found that teachers conducted home visits to explore the reason for the non-attendance, hence playing an important role as change agents.

Drivers

Enrolment

Evidence from the interviews showed that school enrolment was conducted by the means of door-to-door identification by the primary school teachers. All children above five years of age were identified and validated with birth listing done by FHWs. The teachers across the nine villages reported this method to be successful, allowing a recruitment rate of 100%, with no children under 5 years not attending school.

"There are no children who are not in school. All children above 5 years come to school." [School Teacher, Kushalgarh]

In Ghatol, it was reported that some children did not attend governmental school given the existence of a private school in the vicinity of three out of five study villages. This was, however, not the case for Kushalgarh where none of the study villages had access to private schools and all enrolled students attended government schools.

"School enrolment is not a problem in this village. Once a year we do a door to door survey to identify school going children. Some families who can afford to send their children to the private do so, rest send them to the government school." [School Teacher, Ghatol]

For peer review only - http://bmjopen.bmj.com/site/about/guidelines.xhtml

High school enrolment levels across the nine villages studied indicate the potential reliability of schools as an intervention delivery platform. Furthermore, the regularity with which children were found to attend school and the degree of parental participation in school activities are important considerations.

Parental participation in homework

According to the teachers from both blocks, homework was given regularly to reinforce the lessons taught. The response to and success in completing the tasks correctly, however, varied across children in the two study blocks and can broadly be divided into three categories: 1) Children who failed to complete the tasks entirely, 2) Children who completed the tasks, but with some errors and 3) Children who completed the tasks with no errors. The teachers mentioned in the interviews that they believe this difference in performance to be at least partially attributable to the level of engagement of the children's family members.

"Only educated mothers can help. We just tell the child to study." [Mothers, Ghatol and Kushalgarh]

The first category of students was mostly those who stayed with their grandparents or the parents who were illiterate and therefore had little to no support for in their education.

"Most children stay with the grandparents. They do not have any environment for studying at home. Homework is done at schools." [School Teacher, Kushalgarh]

The second category of students were those where the child's mother was educated till 5th standard or had a school going elder sibling who assisted him/her. Occasionally, the parents of children in the third category were engaged in teaching the child, hence leading to better performance in homework tasks. According to teachers in Kushalgarh, the majority of students belonged to the first category and the remaining few were in the second category. In case of Ghatol, the first category of students was relatively smaller. In general, these results suggest that teachers are an important reference point for students and play an essential role in supporting students, especially where limited support is available at home.

For peer review only - http://bmjopen.bmj.com/site/about/guidelines.xhtml

Health and WASH

The school adopted different approaches to improve student health and hygiene practices. For health, an annual check-up was conducted each year by ANMs. The teachers explained that ANMs came with adequate medicine for camps, and mostly provided first aid treatment. They also identified children who needed special medical attention. Health services were also available from their local AWC. The teachers felt this was a successful and necessary initiative, given the generally limited knowledge about health and hygiene in the local community.

Hand pumps available in the premises of most schools, were used as a drinking water source though the water quality was not tested regularly. In a majority of schools, the same hand pumps were also used for handwashing. In a few schools, special filtering plants were present. Toilets were available for children, in schools; while only few had separate facilities for boys and girls. Overall, the toilets in most schools were in an unsatisfactory condition, with a large proportion of toilets dysfunctional.

Several teachers reported the provision of special programmes related to diarrhoea prevention and hygiene promotion in schools. In these programmes, awareness was generated about health and hygiene practices. The teachers felt that children proved to be an effective channel to pass on this information at home, hence fostering better understanding about nutrition, health and hygiene practices, also across generations.

"We tell the students about good hygiene practices like handwashing, taking a bath every day, cutting nails. They then go and tell their family. Spreading messages through children are very effective."
[School Teacher, Ghatol]

For peer review only

Opportunities

Availability of playgrounds and other facilities/ Games

The presence of playgrounds or outdoor activity areas was noted amongst all except two schools visited. Additionally, physical education teachers and sporting facilities were available in a few schools. It was reported that facilities were mostly only operational during summer rather than rain season. In the KIIs, several teachers noted they incorporate play activities in the last period of every school day, since no such opportunities were available at the children's homes. The teachers believe this to be essential for the development of the children, promoting both their physical and mental wellbeing.

For peer review only - http://bmjopen.bmj.com/site/about/guidelines.xhtml

"Playing is very necessary for the holistic development of children. We devote the last period each day for that." [School Teacher, Ghatol]

FGDs showed that not all parents were convinced of the usefulness of play activities for their children.

"Parents sometime question us why we play games, school is for studying not playing." [School Teachers, Kushalgarh]

This highlights another potential area where teachers could use their positive standing in the local community to influence and change current practices.

Mid-day meals

All teachers, PRI members and mothers responded that the mid–day meal was prepared in the school and served regularly to students. The menu for each day was fixed by the Education Department, with diet plans including cereals such as wheat and rice, pulses, vegetables. Fruit was also served once a week. The food was prepared by a cook and an assistant under the supervision of the principal or other teachers.

"I have seen food being served in schools regularly. Sometimes I go to check the quality also." [Ward Panch, Ghatol]

No concerns regarding school meal quality were raised in the FGDs with mothers, nor in the interviews with PRIs. The teachers, especially from schools in Kushalgarh, were of the opinion that nutrition diversity offered in mid-day meals was superior to foods served to children at their homes.

"Yes, we provide food to children regularly. [...] The food that they get in schools is better than what they get in home. Dal is not cooked in the homes every day. Fruit is also an attraction among kids." [School Teachers, Kushalgarh]

Furthermore, it was reported that the general standards of cleanliness and hygiene, e.g. when storing, preparing and serving food, were higher at the schools than those encountered at home: at schools, clean water was always used to prepare and cook meals and hygiene practices were implanted for hands, cooking equipment and cutlery.

For peer review only - http://bmjopen.bmj.com/site/about/guidelines.xhtml

Discussion

Appropriate supplementary Infant and Young Child Feeding (IYCF) are inherently linked with growth and development, especially in children younger than 24 months. Based on the evidence globally, universal coverage of optimal breastfeeding could prevent 13% of global deaths occurring in children less than five years of age, while appropriate IYCF practices could result in an additional 6% reduction in under-five mortality [1].

Efforts to improve IYCF hence remain a potent intervention target. Awareness generation among mothers and families with young children is crucial for successful intervention implementation. Identifying occasions and settings where community members gather at regular intervals hence is a crucial step to increase reach of possible health interventions, especially in rural states.

Since 1986 the World Health Organisation (WHO) has placed particular emphasis on the potential of health promotion strategies [13] in a school-based setting, but the sustainability of school-based interventions has remained relatively unexplored, especially in low- and middle-income countries [14]. Traditionally, the role of schools is to build human capital and social cohesion. During the era of Millenium Development Goals (MDGs) (nowadays known as 'Sustainable Development Goals' (SDGs)), school-enrolment and universal primary education have increased in developing countries, giving school-based interventions the potential to reach significant numbers of children [5]. School infrastructure and physical environment, hygiene campaigns, policies, curricula and teachers have the potential to positively influence physical, socio-cultural, and policy characteristics of the micro-environment [15]. Moreover, schools are well known to be used as a centre of change especially during crises in high-income countries [16] [17] as well as low- and middle-income countries [18] [19].

In our study, schools have been shown to be an important and viable setting for community mobilisation in the context of nutrition, health and hygiene practices. The qualitative evidence from the FDGs and KIIs, as well as intervention development and the piloting of the fore-mentioned interventions, indicate that schools may indeed represent a feasible potential platform for community engagement, and mobilisation. Many existing, local government bodies within the village, e.g. Angawadi Centre, Health Sub centre, Panchayat, Public distribution system, are closely associated with the schools, again rendering them a potentially suitable site for intervention delivery to collaboratively improve IYCF practices.

Enrolment rates were high across the nine villages investigated in this study. Schools and associated staff were dedicated to encouraging parent involvement in the school and discouraging student absenteeism. Initiatives such as the mid-day meal, play opportunities and education on health and hygiene (e.g. through the WASH) all highlight the schools' potential for community engagement and

For peer review only - http://bmjopen.bmj.com/site/about/guidelines.xhtml

reliability as an intervention delivery platform. Festivals and local celebrations such as Janmashtami, Independence Day, Republic Day, Annual Day Celebration take place on the school campuses. Furthermore, schools can act as research sites, playing a crucial role in capturing the diversity and dynamics that exist in particular communities. As one of the only functional places in the rural communities, they represent the 'beating heart' of villages, where community members of all generations gather at frequent intervals.

In this context, teachers were found to be a crucial stakeholder in bringing about positive change in local communities, acting as a reliable and sophisticated source of information regarding the communities they serve alongside being potential change agents for children, their parents and the wider community via; (a) their connections and respect from their local communities and (b) their interest in co-designing interventions with us. Thus, engaging with the schoolteachers in further analysis of the problem and co-designing any future interventions holds merit. This has been recognized locally and was translated into initiatives of the District Collector if Bansawar, including the Swachhta Pakhwada and Alakh programmes where school staff, parents and PRI members gather at regular intervals to discuss challenges and improvement opportunities for the village.

Furthermore, many school-based interventions have the advantage of being a cost-effective method to deliver interventions with a broad reach, enrolling participants from different socio-economic backgrounds, ages and at-risk groups [19]. It is evident from the literature that children have acted as agents of change in multiple health-related community interventions to improve their own and their parents' health [20][21]. Studies have demonstrated that children learn about a health topic and share that information with their parents or their community, thereby fostering an increase in health awareness and health promoting behaviours across generations [22][23]. This combined with children's quick learning ability and regular access to high-quality information through schools and associated staff, gives children the potential to become positive community influencers.

Finally, whilst the findings from our study strongly suggest that schools represent a suitable site for community mobilisation and health promotion in rural India, the possible limitations of our primary data collection need to be considered. One significant challenge was that of time –for efficiency purposes, data collection was restricted to 2 hours per village, hence possibly limiting the information gathered from the study participants in the FDGs and KIIs. Secondly, conducted between January and March 2018, the timing of our primary data collection exercises overlapped with the peak of agricultural season in the Banswara district. This may have impacted our sample size, due to absence of functionaries at schools and/or preoccupation of participants of our household survey with agricultural activities.

In the future, efforts thus need to be focused into gathering data from a larger sample size and area, to capture the complexity of actual practices and hence, gain a more holistic overview of current

For peer review only - http://bmjopen.bmj.com/site/about/guidelines.xhtml

challenges and opportunities. Our qualitative findings will then be able to translated into practice, namely by developing culturally-sensitive, locally-effective, school-based interventions to positively influence in IYCF in rural India. Future studies utilising the PANChSHEEEL model (IYCF intervention package combined with multilevel engagement with schools and AWC at the centre) will investigate this further, and explore how the co-location with the Angawandi centres may be best leveraged in subsequent intervention development and implementation.

Conclusion

Schools are likely to act as a central theme in deployment for behaviour change interventions, owing to the fact that they form a central part of the child's daily routine in addition to schools being a centre of community activities. These activities could be incorporated into the ongoing programmes, through either leveraging of the mid-day meal program or other such child development schemes that will provide opportunities to use these interactions to improve the wider determinants of healthy living in children as well as their families.

Developing a supportive environment around schools, geared towards reinforcing messaging on healthy practices and using children as change agents to bring about community change therefore requires further investigation.

Ethics approval and consent to participate: The PANChSHEEEL Project protocol, including qualitative and quantitative data collection methods as well as the tools obtained ethical approval from the UCL ethics committee (REF NO: 4032/002) in United Kingdom and the Sigma-IRB (REF NO: 10025/IRB/D/17-18 REF NO: 4032/002) in India.

Consent for publication: Not applicable.

Declaration of Conflicting Interests: The authors declared no potential conflicts of interest with respect to the research, authorship, and/or publication of this article.

Data Sharing Statement: The data of this study is available from the corresponding author upon reasonable request.

For peer review only

Funding: This research was supported by the MRC Global Challenges Research Fund [grant number: MR/P024114/1]. Prof Monica Lakhanpaul was supported by the National Institute for Health Research (NIHR) Collaboration for Leadership in Applied Health Research and Care (CLAHRC) North Thames at Bart's Health NHS Trust. Dr. Priti Parikh is currently supported by the BBOXX/Royal Academy of Engineering Senior Research Fellowship.

Author Contributions: ML, MCL, PP, RK, and SS conceived the original concept of the study and designed the research methodology. SR, ML, PraP, RK, HC, SS, SPP, TS, and PP carried out the interviews, analysed the data and contributed to manuscript writing. ML, LM, PP, SR, HC, NS, SS, SPP, LB, VV, PD and RK , interpreted the data, validated the study and revised the manuscript critically for important intellectual content. SA and ICD wrote the manuscript, contributed to the data analysis, edited the final manuscript and prepared for submission. ML had primary responsibility for the final editing of the manuscript and content. All authors read and contributed to reviewing the analysis of the data, the designing of the manuscript, and the approval of the final manuscript.

Acknowledgments: On behalf of the PANChSHEEEL team (Professor Monica Lakhanpaul, Susrita Roy, Dr Lorna Benton, Professor Marie Lall, Rajesh Khanna, Professor Virendra Kumar Vijay, Sanjay Sharma, Dr Logan Manikam, Neha Santwani, Dr Hanimi Reddy, Hemant Chaturvedi, Shereen Allaham, Satya Prakash Pattanaik, Tol Singh, Pramod Pandya, Priyanka Dang, Dr Priti Parikh), we gratefully acknowledge the input from our community champions and field team which made this project possible. We would like to thank our research partners; Madhav Foundation (Ritu Chhabria & Ritu Prakash), EKAM Foundation (Dr.Sai Lakshmi, Ms. Neeta Karal Nair, Mr. Manoharan, Dr. Sathya Jegannathan and Ms. Benita), Foundation for Research in Community Health India (Dr Nerges Mistry & Dr. Shilpa Karvande), Society for Nutrition Education & Health Action (SNEHA; Dr Nayreen Daruwalla and Dr David Osrin) and our technical advisory board (Prof Anita Saxena, Mr Anand Karve, Prof V M Chariar, Prof T. Sundararaman, Dr Ian Warwick, Dr Ramesh Mehta, Prof Atul Singhal, John Pelton, Professor Sachin Maheshwari, Dr Sofia Strummer, Mr Himanshu Parikh & Dr Amita Kashyap) for their input throughout the study. The opinions in this paper solely reflect the views of the authors, not of organisations who financially supported this research. All data used in this research is the property of the authors. It is only obtaineable by email from the corresponding author.

For peer review only - http://bmjopen.bmj.com/site/about/guidelines.xhtml

References

[1] World Health Organisation. Infant and Young Child Feeding: Model Chapter for Textbooks for Medical Students and Allied Health Professionals. SESSION 1: The importance of infant and young child feeding and recommended practices. Geneva, 2009. Available from: https://www.ncbi.nlm.nih.gov/books/NBK148967/

[2] Mitra M, Kumar PV, Chakravarty S, Bharati P. Nutritional status of Kamar tribal children in Chhattisgarh. Indian J Pediatric 2007, 74(4); 381-4.

[3] Chakrabarti S, Raghunathan K, Alderman H, Menon P, Nguyen P. India's Integrated Child Development Services programme; equity and extent of coverage in 2006 and 2016. *Bull World Health Organ*. 2019;97(4):270-282. doi:10.2471/BLT.18.221135

[4] Mukamana O, Johri M. What is known about school-based interventions for health promotion and their impact in developing countries? A scoping review of the literature. *Health Education Research*, Volume 31, Issue 5, October 2016, Pages 587–60. Available from: https://doi.org/10.1093/her/cyw040 [Accessed: 12/09/20]

[5] Bresee S, Caruso BA, Sales J, Lupele J, Freeman MC. 'A child is also a teacher': exploring the potential for children as change agents in the context of a school-based WASH intervention in rural Eastern Zambia. *Health Education Research*, Volume 31, Issue 4, August 2016, Pages 521–534.

[6] Swarup A, Srivastava R. WCD [Internet]. Wcd.nic.in. 2017 [cited 28 October 2021]. Available from: https://wcd.nic.in/sites/default/files/Co-location%20of%20AWCs%20in%20School%20Premises.pdf

[7] Parikh, P., Kwami, C.S., Khanna, R., Lall, M., Reddy, H., Benton, L., Sharma, S., Vijay, V.K., Manikam, L. and Lakhanpaul, M., 2021. Linkages between environmental factors (WASH and energy) and Infant and Young Child Feeding practices in rural India: implications for cross-sectoral interventions for child health. *Journal of Water, Sanitation and Hygiene for Development*

[8] Lakhanpaul M. Integrated Health, education and environmental (HEE) intervention to optimise infant feeding practices through schools and Anganwadi networks in India. UK Research and Innovation. 2017. Available from: https://gtr.ukri.org/projects?ref=MR%2FP024114%2F1

For peer review only - http://bmjopen.bmj.com/site/about/guidelines.xhtml

[9] Parikh P, Kwami C, Khanna R, Lall M, Reddy H, Benton L et al. Linkages between environmental factors (WASH and energy) and Infant and Young Child Feeding practices in rural India: implications for cross-sectoral interventions for child health. IWA Publishing. 2021. Available from: https://iwaponline.com/washdev/article/doi/10.2166/washdev.2021.005/83444/Linkages-between-environmental-factors-WASH-and

[10] PANChSHEEEL Project - University College London. Pahus.org. 2021. Available from: https://www.pahus.org/panchsheeel-project-

[11] Seale C. Quality in Qualitative Research - Clive Seale, 1999 [Internet]. SAGE Journals. 1999. Available from: https://doi.org/10.1177/107780049900500402

[12] Braun V, Clarke V. Using thematic analysis in psychology [Internet]. Taylor & Francis. 2008 [cited 29 October 2021]. Available from: https://www.tandfonline.com/doi/abs/10.1191/1478088706qp063oa

[13] World Health Organisation (WHO). Ottawa Charter for Health Promotion. Ottawa, 1986. Available from: http://www.euro.who.int/__data/assets/pdf_file/0004/129532/Ottawa_Charter.pdf?ua=1

[14] Herlitz L, MacIntyre H, Osborn T et al. The sustainability of public health interventions in schools: a systematic review. Implementation Sci 15, 4 (2020). Available from: https://doi.org/10.1186/s13012-019-0961-8

[15] Brown T, Summerbell C. Systematic review of school-based interventions that focus on changing dietary intake and physical activity levels to prevent childhood obesity: an update to the obesity guidance produced by the National Institute for Health and Clinical Excellence. Obes Rev 2009;

[16] Khambalia AZ, Dickinson S, Hardy LL, Gill T, Baur L. A synthesis of existing systematic reviews and meta-analyses of school-based behavioural interventions for controlling and preventing obesity. Obes Rev 2012; 13:214–33.

[17] Li M, Li S, Baur LA, Huxley RR. A systematic review of school-based intervention studies for the prevention or reduction of excess weight among Chinese children and adolescents. Obes Rev 2008; 9:548–59.

For peer review only - http://bmjopen.bmj.com/site/about/guidelines.xhtml

[18] Ribeiro IC, Parra DC, Hoehner CM, Soares J, Torres A, Pratt M, Legetic BD, Malta DC, Matsudo VR, Ramos LR, et al. School-based physical education programs: evidence-based physical activity interventions for youth in Latin America. Glob Health Promot 2010; 17:5 –15.

[19] Verstraeten R, Roberfroid D, Lachat C, Leroy JL, Holdsworth M, Maes L, Kolsteren PW. Effectiveness of preventive school-based obesity interventions in low- and middle-income countries: a systematic review. Am J Clin Nutr. 2012 Aug;96(2):415-38. doi: 10.3945/ajcn.112.035378. Epub 2012 Jul 3. PMID: 22760565.

[20] Onyango-Ouma, W. (2003). Children as partners in health communication in a Kenyan community. Anthropology in Action, 10(1), 25-33.

[21] Sedighi I, Nouri S, Sadrosadat T, Nemati R, Shahbazi, M. Can children enhance their family's health knowledge? An infectious disease prevention program. Iran J Pediatr, 22(4), 493-498.

[22] Gadhoke P, Christiansen K, Rowan M, Aniliker J, Frick K, Gittelsohn, J. Formative research supports a change agent approach to prevent obesity in American Indian households. The FASEB Journal, 26(1). 2012. doi: 10.1096/fasebj.26.1_supplement.389.7

[23] Mosavel M, Genderson MW. Daughter-initiated cancer screening appeals to mothers. J Cancer Educ, 31(4), 767-775. 2016. doi:10.1007/s13187-015-0949-2

For peer review only

For peer review only - http://bmjopen.bmj.com/site/about/guidelines.xhtml

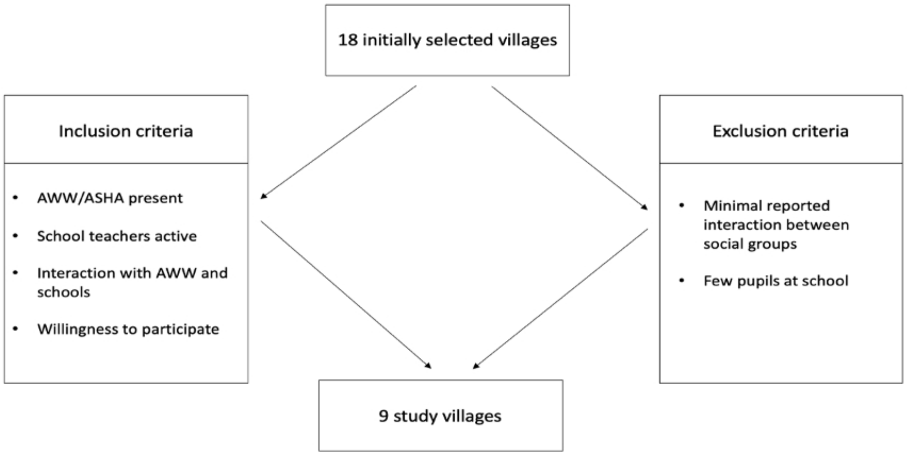

Figure 1. Inclusion and exclusion criteria for the study villages and schools

159x80mm (150 x 150 DPI)

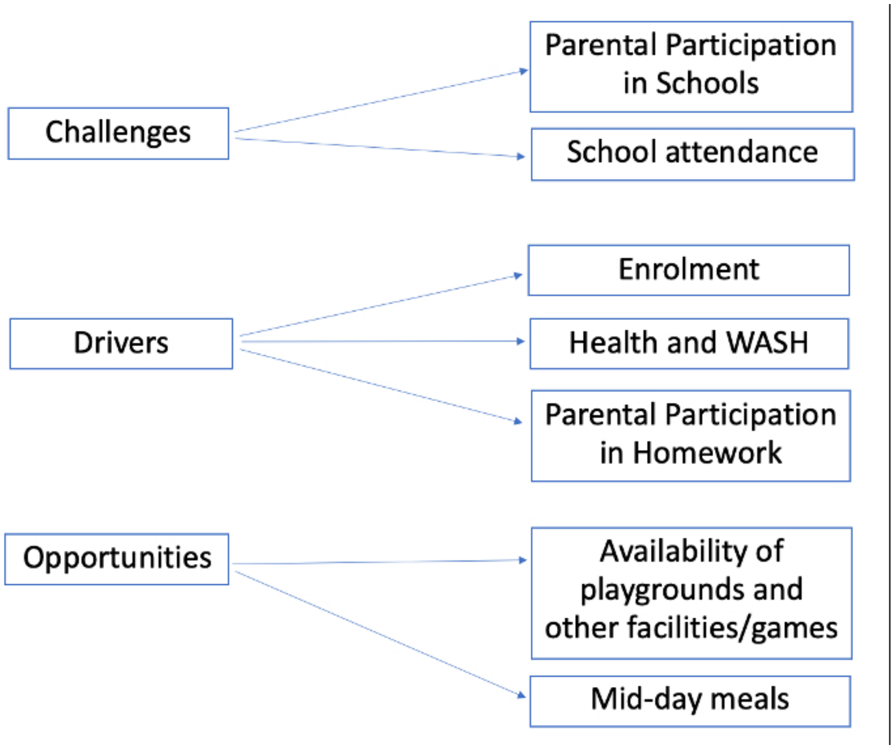

Figure 2. Overarching themes identified during the thematic analysis of KIIs and FDGs: challenges, drivers, and opportunities.

129x95mm (150 x 150 DPI)

Manuscript: Title: The Role of Schools in the PANChSHEEEL Project – an Approach to Identify and Address Factors Influencing Infant and Young Child Feeding Practices and Nutrition in India.

Consolidated criteria for reporting qualitative studies (COREQ): 32-item checklist

Developed from:
Tong A, Sainsbury P, Craig J. Consolidated criteria for reporting qualitative research (COREQ): a 32-item checklist for interviews and focus groups. *International Journal for Quality in Health Care*. 2007. Volume 19, Number 6: pp. 349 – 357

For peer review only

No. Item	Guide questions/description	Reported on Page #
**Domain 1: Research team and reflexivity**		
Personal Characteristics		
1. Interviewer/facilitator	Which author/s conducted the interview or focus group?	Page 7-9
2. Credentials	What were the researcher's credentials? E.g. PhD, MD	Page 7 & 8
3. Occupation	What was their occupation at the time of the study?	Page 7 & 8
4. Gender	Was the researcher male or female?	Page 7 & 8
5. Experience and training	What experience or training did the researcher have?	Page 8
Relationship with participants		
6. Relationship established	Was a relationship established prior to study commencement?	Page 6
7. Participant knowledge of the interviewer	What did the participants know about the researcher? e.g. personal goals, reasons for doing the research	Page 6-8
8. Interviewer characteristics	What characteristics were reported about the interviewer/facilitator? e.g. Bias, assumptions, reasons and interests in the research topic	Page 6

**Domain 2: study design**		
Theoretical framework		
9. Methodological orientation and Theory	What methodological orientation was stated to underpin the study? e.g. grounded theory, discourse analysis, ethnography, phenomenology, content analysis	Page 2 & 6
Participant selection		
10. Sampling	How were participants selected? e.g. purposive, convenience, consecutive, snowball	Page 8
11. Method of approach	How were participants approached? e.g. face-to-face, telephone, mail, email	Page 6-8
12. Sample size	How many participants were in the study?	Page 7, 9 & 10
13. Non-participation	How many people refused to participate or dropped out? Reasons?	Page 7
Setting		
14. Setting of data collection	Where was the data collected? e.g. home, clinic, workplace	Page 8
15. Presence of non-participants	Was anyone else present besides the participants and researchers?	Page 8
16. Description of sample	What are the important characteristics of the sample? e.g. demographic data, date	Page 6 & 8
Data collection		
17. Interview guide	Were questions, prompts, guides provided by the authors? Was it pilot tested?	Page 9
18. Repeat interviews	Were repeat interviews carried out? If yes, how many?	N/A
19. Audio/visual recording	Did the research use audio or visual recording to collect the data?	Page 9
20. Field notes	Were field notes made during and/or after the interview or focus group?	Page 9
21. Duration	What was the duration of the interviews or	N/A.

For peer review only

	focus group?	
22. Data saturation	Was data saturation discussed?	Page 8 & 9
23. Transcripts returned	Were transcripts returned to participants for comment and/or correction?	Page 9
**Domain 3: analysis and findings**		
Data analysis		
24. Number of data coders	How many data coders coded the data?	Page 9
25. Description of the coding tree	Did authors provide a description of the coding tree?	No.
26. Derivation of themes	Were themes identified in advance or derived from the data?	Page 10-14
27. Software	What software, if applicable, was used to manage the data?	Page 9
28. Participant checking	Did participants provide feedback on the findings?	Page 9
Reporting		
29. Quotations presented	Were participant quotations presented to illustrate the themes/findings? Was each quotation identified? e.g. participant number	Yes.<br>Page 10-14
30. Data and findings consistent	Was there consistency between the data presented and the findings?	Yes.<br>Page 10-14
31. Clarity of major themes	Were major themes clearly presented in the findings?	Yes.<br>Page 10 & 13
32. Clarity of minor themes	Is there a description of diverse cases or discussion of minor themes?	Yes<br>Page 10-14

For peer review only
